# Common Feeding Practices Pose A Risk to the Welfare of Horses When Kept on Non-Edible Bedding

**DOI:** 10.3390/ani10030411

**Published:** 2020-03-02

**Authors:** Miriam Baumgartner, Theresa Boisson, Michael H. Erhard, Margit H. Zeitler-Feicht

**Affiliations:** 1 Ethology, Animal Husbandries and Animal Welfare Research Group, Chair of Organic Agriculture and Agronomy, TUM School of Life Sciences Weihenstephan, Technical University of Munich; Liesel Beckmann-Str. 2, 85354 Freising, Germany; 2 Chair of Animal Welfare, Ethology, Animal Hygiene and Animal Husbandry, Department of Veterinary Sciences, Faculty of Veterinary Medicine, Ludwig-Maximilians-University Munich, Veterinärstr. 13, 80539 Munich, Germany

**Keywords:** horse behaviour, feed intake pause, bedding, welfare indicator, feeding practices, roughage, horse welfare, individual housing system

## Abstract

**Simple Summary:**

It is a basic high priority need of every horse to take in roughage continuously. In order to ensure the horses’ behavioural, physical and mental welfare, any pause of feed intake should not last for more than 4 hours. However, this basic need is often neglected in practice. The aim of the presented study was to assess the welfare of horses that are fed restrictively (*non ad libitum*) and kept in individual housing systems. We analyzed whether the feed intake behaviour of horses on edible bedding differs from the one of horses on non-edible bedding. As a common practice, the individually stabled horses were fed roughage twice or thrice a day. Our results showed that with this restrictive feeding practice, the horses were not able to eat any roughage for approx. 9 h during the night. Horses on non-edible bedding altered their feed intake behaviour - i.e., they paused less often during their meals and at a later point in time than the horses on edible bedding. We conclude that special feeding patterns have to be implemented (e.g., automated forage feeding systems) to avoid any impairment of the horses’ welfare if kept on non-edible bedding.

**Abstract:**

During the evolution of the horse, an extended period of feed intake, spread over the entire 24-h period, determined the horses’ behaviour and physiology. Horses will not interrupt their feed intake for more than 4 h, if they have a choice. The aim of the present study was to investigate in what way restrictive feeding practices (*non ad libitum*) affect the horses’ natural feed intake behaviour. We observed the feed intake behaviour of 104 horses on edible (*n* = 30) and non-edible bedding (*n* = 74) on ten different farms. We assessed the duration of the forced nocturnal feed intake interruption of horses housed on shavings when no additional roughage was available. Furthermore, we comparatively examined the feed intake behaviour of horses housed on edible versus non-edible bedding. The daily restrictive feeding of roughage (2 times a day: *n* = 8; 3 times a day: *n* = 2), as it is common in individual housing systems, resulted in a nocturnal feed intake interruption of more than 4 hours for the majority (74.32%, 55/74) of the horses on shavings (8:50 ± 1:25 h, median: 8:45 h, minimum: 6:45 h, maximum: 13:23 h). In comparison to horses on straw, horses on shavings paused their feed intake less frequently and at a later latency. Furthermore, they spent less time on consuming the evening meal than horses on straw. Our results of the comparison of the feed-intake behaviour of horses on edible and non-edible bedding show that the horses’ ethological feeding needs are not satisfied on non-edible bedding. If the horses accelerate their feed intake (also defined as “rebound effect”), this might indicate that the horses‘ welfare is compromised. We conclude that in addition to the body condition score, the longest duration of feed intake interruption (usually in the night) is an important welfare indicator of horses that have limited access to roughage.

## 1. Introduction

Under natural conditions horses spend most of their time foraging and grazing, approximately 12 to 16 h of the 24-h-period are is spent on the ingestion of food [1,2,3,4,5,6,7,8]. Approximately 60%–70% of the daytime and 30%-40% of the night time is spent on feed intake [7,8,9,10,11]. Even when stabled, horses which are fed *ad libitum* divide their feed into approximately 10 meals, comparable to free-ranging horses [1,4,11,12,13,14,15,16,17,18]. Neither during the day, nor at night do horses pause voluntarily for longer than 3 to 4 hours between meals, nor do they fast [1,2,3,6,7,8,11,12,13,15,18,19,20,21,22]. The interruptions in feed intake are mainly due to the horse’s need to engage in other behaviours, e.g., resting, interacting with social partners and comfort activities. Resting periods alternate with feed intake [7,8,20,21,22,23]. Therefore, other motivations must overrule the horses´ constantly activated motivation to forage before horses interrupt feeding.

Contrary to popular belief, a feeling of satiation or myofibrillary fatigue of the masticatory muscles does not limit the horses in their intake of feed [2,7,8,20,24,25]. The ability to consume high amounts of roughage and thus the high motivation to eat is evolutionary. This is due to the fact that for thousands of years, the vast steppes of Eurasia have offered a wide plant diversity, yet scant vegetation. Horses are adapted to a diet that is rich in structural fibres and low in energy (i.e., rapidly hydrolyzable carbohydrates) [7,8,18,26]. Due to the horses´ evolutionary fitness benefit (“niche construction theory”), they have the ability to forage an unlimited amount of low energy and high fibre food (ultimate behavioural control mechanism) [27]. If there is an energy deficit, metabolic and gastrointestinal cues as well as external stimuli (e.g., food supply) will increase the horses´ motivation to eat [28]. It is obvious that only nursing foals limit their feed intake when enough energy has been taken in. In adult horses, this capability is diminished. The horse does not recognize when the energy intake exceeds the horse’ demand, especially when eating energy-rich, low-fibre food [8,20]. Hence, a continuous intake of food is a high priority basic need for horses and that irrespective of the current energy demand. If horses are fed in individual housing systems according to common feeding practices, the horses’ natural feed intake behavior can be compromised [5,7,8].

A large number of studies have shown that poor feeding practices can lead to health problems in the digestive tract of horses. In particular, long pauses of feed intake are associated with colics caused by dysfermentation [29,30,31], the development of stomach ulcers [32,33,34,35,36,37,38] and constipation [39,40]. According to Luthersson et al. [37], the risk of grade II gastric ulcers (equine gastric ulceration syndrome severity score ≥2) is significantly increased if the feed intake pause lasts for 6 h or longer. The explanation for this is a decreased production of saliva and a reduced buffering capacity in the stomach due to a lack of roughage [31].

In addition, if horses are restrictively fed with hay (*non ad libitum*) horses kept on shavings show abnormal repetitive behaviour more often than horses kept on straw [41,42]. According to Marsden [43], with horses that spend a shorter time on eating, there is a higher risk that they show abnormal behaviour for longer. Lack of roughage is considered as one of the main causes of behavioural disorders in horses [7,8,42,43,44,45,46,47,48,49,50,51]. However, a direct connection between the behavioural disorder “crib-biting” in horses and the occurrence of stomach ulcers has not been proven. This indicates that both diseases result from housing and management related stress and are not interdependent [52,53]. Consequently, horses have evolved in a way that requires continuous food intake, from an ethological (natural behaviour) and a physiological (digestive system) point of view. According to Fraser [54] three different, yet overlapping aspects need to be considered to evaluate animal welfare: basic health and functioning, natural living (behaviour) and affective states. If the horses´ foraging periods are too short or the feed intake pauses too long, the horses are not able to express their natural behaviour. According to the German “Guidelines for Good Animal Welfare Practice for the Keeping of Horses” of the German Federal Ministry of Food and Agriculture (GFFA, 55) a minimum feeding period of 12 h as well as a maximum of 4 hours of feed intake pauses is required to avoid compromises in animal welfare. Studies carried out more than 30 years ago have already shown that if horses are kept under common husbandry conditions with restrictive hay feeding on non-edible bedding, the time budget of the horses deviates considerably. Instead of the natural feeding period of approximately 12 h per day, the horses are sometimes only able to consume food for approximately 4 hours of the 24-h day [5]. Horses kept on non-edible bedding such as wood shavings are not able to obtain roughage apart from the hay meals that they are fed twice or thrice during daytime. Hence, there is a risk that the maximum feed intake pause of 4 hours will be exceeded [6,7,55]. This applies in particular to the nocturnal feed intake interruption. To date, no studies on feed intake pauses of horses kept in individual housing systems have been published. However, this is an important research topic because the individual housing system is currently the most common method of keeping horses in Europe. It is evident that straw, as a source of roughage, has benefits for the horses´ welfare. If straw is used as a bedding material, the horses engage with it in a higher frequency and duration. In particular, the horses eat the bedding and spend less time on inter alia standing motionlessly [56,57]. Since engagement is one of the most important functions that bedding material is supposed to fulfill, Werhahn et al. [56] recommend to use straw over straw pellets and shavings to fulfill behavioural needs. Moreover, the result of a current study is that roughage significantly influences the horses´ welfare if they are housed individually. If hay is fed restrictively, straw as bedding prevents dietary deprivations of horses [58].

It is still unknown whether the natural feed intake pauses of horses on non-edible bedding that are fed restricted amounts of hay are exceeded. The satisfaction of this basic need could potentially be restricted. The aim of the present study was to investigate in what way restrictive feeding practices alter the horses’ natural feed intake behaviour and thereby compromise the horses´ welfare. In particular, the study’s aim was to i) determine the duration of the nocturnal feed intake interruption of horses housed on shavings when no additional roughage was available and ii) examine the feed intake behaviour of horses housed on edible versus non-edible bedding comparatively. We mainly focused on the time the horses needed to finish the evening meal, the duration and frequency of pauses during feed intake and at what time a pause occurred first (latency) in general and per category according to their duration.

## 2. Materials and Methods

### 2.1. Animals

The study took place on 10 different farms in the greater Munich (Germany) area and included a total of 116 sport and leisure horses of different race, age and sex. Only horses that did not suffer from any gastrointestinal disease or teeth problems (according to the farm managers) were included in the study. Ponies were explicitly excluded. Hence, 104 of the 116 horses were included in the study. They were all crossbred horses. All of these selected horses had a normal nutritional status (body condition score 5 or 6 on a scale from 1 to 9 [59,60]). Of the 104 horses, 34 were housed in individual boxes without the possibility of putting their head outside (grilled partition between and in the front of the boxes), 25 were housed in individual boxes with the possibility of putting their head outside (open window toward the external environment) and 45 of the horses were housed in individual boxes having direct access to a small outside yard (without vegetation) of the approximate size of the box (12–15 m^2^). All horses had at least the possibility of visual contact with conspecifics (limited tactile contact). During the study period, every horse that was kept in an individual housing system was allowed to graze for approximately 6 hours daily (4–8 h). At least 2 hours before the feeding of the evening meal all horses had to be back in their individual boxes. On every farm the horses were either stabled on edible or on non-edible bedding material.

### 2.2. Behaviour Observation

Different direct behaviour observations were performed on a different number of subsamples. The selection of subsamples was based on the following precondition: Horses stabled side by side on edible or non-edible bedding in boxes that are easily visible to the observer (64 out of 104 horses). These horses were continuously observed during the evening feeding (“continuous behaviour sampling”, Table 1) [61]. The remaining 40 horses were all stabled on non-edible bedding and were sampled discontinuously from the time of the beginning to the end of the evening roughage meal. The observations were carried out between 2:30 p.m. and 6:00 p.m., depending on the farm, and lasted until midnight at the latest.

Subsequently, the voluntarily taken pause of feed intake will be defined as the “feed intake pause” and the forced interruption of feed intake due to restrictive roughage supply will be defined as the “feed intake interruption”. We observed these horses continuously and determined the time it took them to finish their evening meal and for how long the feed intake interruption lasted during the night (55 Continuously Observed (CO-) horses and nine Discontinuously Observed (DCO-) horses = 64, Table 1). In addition, we continuously observed the same 55 out of these 64 horses to assess the duration of feed intake pauses during the evening meal (CO-horses, *n* = 55). It was impossible to continuously observe the remaining nine horses as they temporarily left the stable for equestrian use during the observation periods (DCO-horses, *n* = 9). For the latter, it was therefore only possible to determine the time they needed to finish the evening meal and the duration of the feed intake interruption during the night.

In addition, the time of beginning and end of the evening roughage meal were recorded for another 40 horses on non-edible bedding (e.g., shavings), which were not directly visible from the observation site (Additionally Recorded (AR-) horses to assess the duration of the nocturnal feed intake interruption, Table 1). We chose to proceed this way in order to include a higher number of horses on non-edible bedding to further strengthen the validity of our study on nocturnal feed intake interruptions. We checked upon the AR-horses regularly but just briefly, to record the time at which they had finished their evening meal. This required only a few seconds and we integrated the results into the overall study. Nine horses (DCO-horses) were not observed continuously, however, both groups of observed horses were roughly of the same sample size (relatively balanced number of horses per topic).

Of the continuously observed (CO-) horses, 30 horses were kept on non-edible bedding (shavings) and 25 horses on edible bedding (straw) (Table 1 and Table 2). The latter had roughage *ad libitum* (hay, straw) at their disposal. The horses were able to eat straw whenever they wanted. The horses kept on non-edible bedding (shavings) did not have unlimited access to feed between roughage meals. All horses were fed with hay (*n* = 9 farms) or hay mixed with straw (*n* = 1 farm) either two (*n* = 8 farms) or three (*n* = 2 farms) times per day.

The same observer observed all of the horses in the identical manner during one evening feeding. The observation was not repeated. The horses had the chance to accustom themselves with the observer for about 1 hour before the observation started. The horses in the study were used to humans and no longer noticed the observer once they had gotten accustomed to his presence. The observer was able to observe a group of 4 to 10 horses simultaneously from one observation point (CO- and DCO-horses). Since we only observed whether the horses took in food, or not, it was possible to distinguish the two activities in up to 10 horses while observing them at the same time (Table 2 as well as Tables S1–S5 in the Supplementary Materials).

### 2.3. Data Sampling

As per definition, the feed intake pause started when the horse had stopped foraging and chewing for at least one minute. The end of the feed intake pause was defined as the time when the horse started to eat again. We used the Microsoft Excel Programme 2016 to calculate the total number of minutes between these two events. Following the example of Krull [4], feed intake pauses were classified in three categories according to their duration: short feed intake pause = 1-10 min, medium feed intake pause = 11-30 min and long feed intake pause = 31 min and longer. For the purpose of our study the presence of isolated stalks of hay on the ground indicated that the horse had finished its evening meal. The time at which each horse finished its evening meal was recorded and defined as the start of the nocturnal feed intake interruption. Horses on straw or horses on non-edible bedding, which had access to straw stalks from the neighbouring box, occasionally started to forage straw after finishing the evening meal. This was not recorded separately. As per our definition, once the horses had finished their evening hay meal the nocturnal feeding interruption started. Each farm manager told us at what time the morning feeding would begin. We used this information as a basis to calculate the duration of the nocturnal feed intake interruption, which lasted from the end of the evening meal to the beginning of the morning feeding. The duration of the nocturnal feed intake interruption was only relevant for horses kept on non-edible bedding (*n* = 74 horses on shavings). We were also able to determine the duration of the nocturnal feed intake interruption for those horses that were temporarily taken out of the box for equestrian use, since all of them had finished their meal by the end of the observation period (*n* = 4 horses on shavings out of 9 Discontinuously observed (DCO-) horses, Table 1).

The observations ended with the end of the evening meal. All horses were observed until they finished their evening meal or until midnight when the stables were closed. The farm managers asked us to leave at midnight, also on those farms where the horses had not finished their evening meal by that time. For both, horses on straw and on shavings (*n* = 55, Continuously Observed (CO-) horses, Table 1 and Table 2), it was recorded when the first feed intake pause was taken in voluntarily (latency) as well as the frequency and the duration of the pauses. The term latency describes the time spent on foraging from the beginning of the evening feeding until the start of the first feed intake pause for each category as mentioned above (short, medium, and long feed intake pauses). Our aim was to find out in what way the duration of feed intake pauses differed when horses were kept on shavings versus horses that were kept on straw. For this purpose we additionally measured the frequency of feed intake pauses for each feeding pause category (*n* = 55 CO-horses, Table 2). The horses whose natural feed intake behaviour could have been altered due to equestrian use and which were thus forced to pause their feed intake during the observation period were excluded (*n* = 9 DCO-horses). We defined the total time until the end of the evening meal as the time, which the 64 horses spent on eating their evening meal or as the time in which hay was available (all CO- and DCO-horses). Every horse started the evening meal at an individual point of time and for all horses the evening meal ended when no more hay was available. As a consequence, the total time the horses spent on eating the evening meal includes the time they spent on feed intake pauses. In the case of DCO-horses, we did not subtract the time the horses spent on equestrian use to not alter the realistic total time that food was available every day.

We feared that a weighing of each prepared evening hay meal might introduce an undesired selection bias of the feeding farmers or caretakers. The farmers were not provided with any details about the study. However had they known that the weight of hay was of relevance for us, they might have altered the amount of hay (deliberately or undeliberately). The farmers or caretakers did not weigh the evening hay meal, thus we also refrained from doing so. For this reason, we did not determine the exact feed intake rate of the horses in the present study.

### 2.4. Statistical Analysis

Descriptive statistics were expressed as mean, standard error of the mean, median, minimum and maximum. Tables and graphs were created in Microsoft Excel Programme 2016. We performed the statistical analysis using Generalized Linear Mixed Models (GLMM). For this, the program R was used (R Studio Version 1.0.143; R software version 3.5.1, R Development Core Team, Vienna, Austria, 2018, package “glm2”) [62]. Six separate models with different dependent variables were calculated: 1) total time for finishing the evening meal, 2) duration of nocturnal feed intake interruption, 3) frequency of feed intake pauses, 4) latency until the first feed intake pause, 5) number of horses that took no feed intake pause in relation to all observed (CO and DCO)-horses per farm, 6) duration of feed intake pause. Outcome variables were treated as continuous variables. We considered the bedding material (explanatory variable) as a categorical factor (straw versus shavings). Explanatory variable was the type of bedding in four models. The farm was specified as a random effect in all models except for those two that related to the effect of the farm as such. It was treated as an explanatory variable in the model in which we tested the effect of the farm on the duration of the nocturnal feed intake interruption and the number of horses that took no feed intake pause in relation to all observed horses per farm. Thus, an effect of feeding practices of the different farms on the nocturnal feed intake interruption was analyzed. The significance level was set at 0.05.

### 2.5. Ethics Statement

This study was non-invasive. It consisted in observing the horses under their current conditions of life. No specific treatments or interventions were done on the animals. The study complies with the Guidelines for Ethical Treatment of Animals in Applied Animal Behaviour and Welfare Research (ISAE Ethics Committee, 2017).

## 3. Results

### 3.1. Total Time for Finishing the Evening Meal

**Figure 1:**
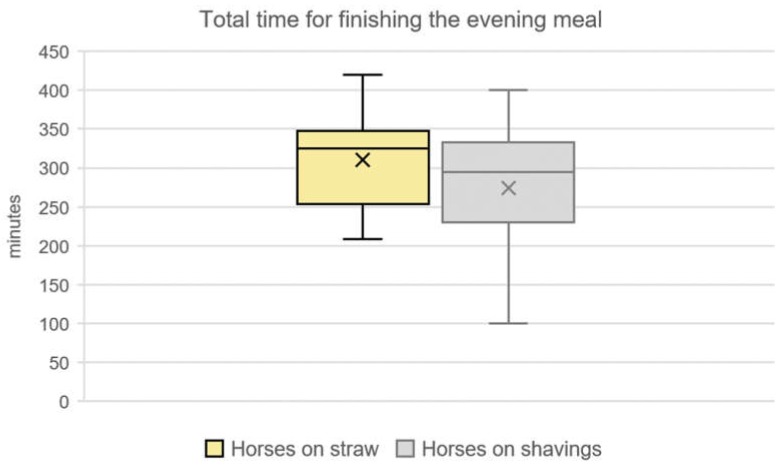
Total time for finishing the evening meal of horses on straw in comparison to horses on shavings, which finished their evening meal within the observation period (79.69%; *n* = 51 out of 64 horses; median: 325 min of *n* = 17 horses on straw; median: 307 min of *n* = 34 horses on shavings, *p* = 0.04).

The evening meal started with the provision of roughage, which was delivered between 3:30 p.m. and 7:00 p.m., depending on the farm. The total time horses needed for finishing the evening meal (CO- and DCO-horses) including feed intake pauses and interruptions for equestrian use was 286 ± 70 min (4:48 h; median: 307 min; minimum: 100 min; maximum: 420 min, *n* = 51 horses). We only included those horses into the analysis, which had finished their evening meal by the end of the observation period (midnight). This applied to the majority of the groups on both bedding materials (*n* = 51/64 CO- and DCO-horses on eight farms, 79.69%). The type of bedding had an influence on the total time for finishing the evening meal (*p* = 0.04, Figure 1). The farm itself was irrelevant for the present study as in all cases the farm´s feeding practices did not have an influence on the total time the horses needed to finish the evening meal on both bedding materials (*p* = 0.13).

### 3.2. Duration of the Nocturnal Feed Intake Interruption

**Figure 2:**
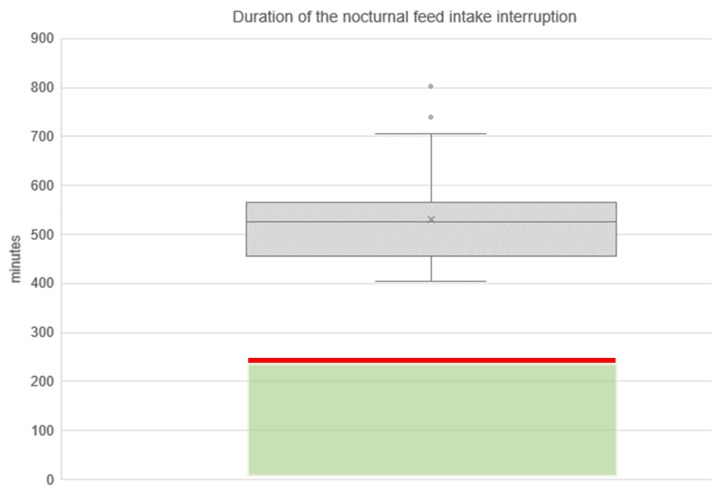
Duration of the nocturnal feed intake interruption of horses on shavings, which had finished their evening meal within the observation period (74.32%; *n* = 55 horses on shavings out of 74 horses on shaving in total). Green box: period without food supply which is still appropriate in terms of welfare; red line: tolerable maximum duration of feed intake interruptions at 240 min (4 hours) according to the German welfare standard [55]).

In contrast to the horses on straw, which had permanent access to roughage since they were kept on edible bedding, the majority of the horses on shavings (74.32%, *n* = 25 CO + 4 DCO-horses + 26 AR-horses = 55 of 74 horses) were forced to interrupt their feed intake during the night for more than 4 hours. The nocturnal feed intake interruption was defined as the period between the end of the evening meal and the beginning of morning feed. The nocturnal feed intake interruption lasted on average 530 ± 85 min (8:50 ± 1:25 h, median: 525 min, minimum: 405 min, maximum: 803 min, Figure 2). The longest feed intake interruption was observed in a horse on shavings, which was fed at 3.30 p.m. and had eaten up after 127 min (2:07 h)—hence it had no roughage available. Thus, this horse was forced to interrupt its feed intake during the night for 13:24 h (803 min). The morning feeding started between 05:45 and 07:15 a.m., depending on the farm.

In 2 of the 10 farms, all seven of the seven CO-horses still had roughage available at the end of the observation period (midnight) (three of those horses on shavings) irrespective of the type of bedding they were housed on. Of the CO- and DCO-horses on shavings (*n* = 34, *n* = 8/10 farms), horses on shavings on three different farms did not finish their evening meal before the observation period ended (midnight), but still had hay available (estimated value: haystack for approximately 1:30 h of feed intake time). Thus, 85.29% (*n* = 29/34) of the sample of CO- and DCO-horses housed on shavings were forced to interrupt their feed intake for more than 4 hours in the night. In the sample of AR-horses on shavings, 65.00% (26 out of 40) had finished their entire roughage meal by midnight. The farms tended to influence the duration of the nocturnal feed intake interruption, but not significantly (*p* = 0.06). This is an indication that the feeding practices on the different farms (time of the evening feeding and amount of provided hay) varied.

### 3.3. Frequency of Feed Intake Pauses and Latency until the First Feed Intake Pause

**Figure 3:**
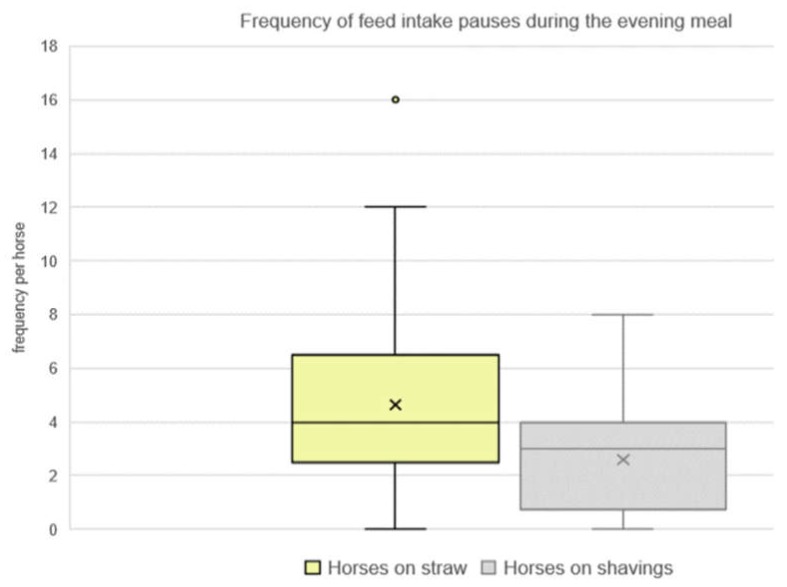
Frequency of feed intake pauses during the evening meal of horses on straw compared to horses on shavings (*n* = 55 horses; median: 4.0 of *n* = 25 horses on straw; median: 3.0 of *n* = 30 horses on shavings, *p* = 0.01)

Horses on straw paused their feed intake more often than horses on shavings (*n* = 25 horses, mean: 4.6 ± 3.7; median: 4.0 compared to n = 30 horses, mean: 2.6 ± 2.2; median 3.0; *p* = 0.01; Figure 3). The farms’ feeding practice tended to have an effect on the frequency of the feed intake pauses (*p* = 0.08).

We calculated the latency until the first feed intake pause irrespective of the pause category. The latency of horses on straw was observed at the same time as for the horses on shavings (*n* = 25 horses, first feed intake pause according to median: 104.00 min, mean: 125 ± 91 min versus n = 30 horses, first feed intake pause according to median: 134 min, mean: 153 ± 81 min, n.s. with *p* = 0.22; no influence of the farm *p* = 0.42). However, two horses on straw (8.00%, *n* = 2/25) of the CO-horses did not pause during the course of the evening meal, whereas seven horses on shavings (23.33%, *n* = 7/30, n.s. with *p* = 0.12) did not take a single feed intake pause. These horses on shavings continued eating hay without any interruption or pause until they were forced to pause when they had finished their evening meal.

### 3.4. Duration of Feed Intake Pause

**Figure 4:**
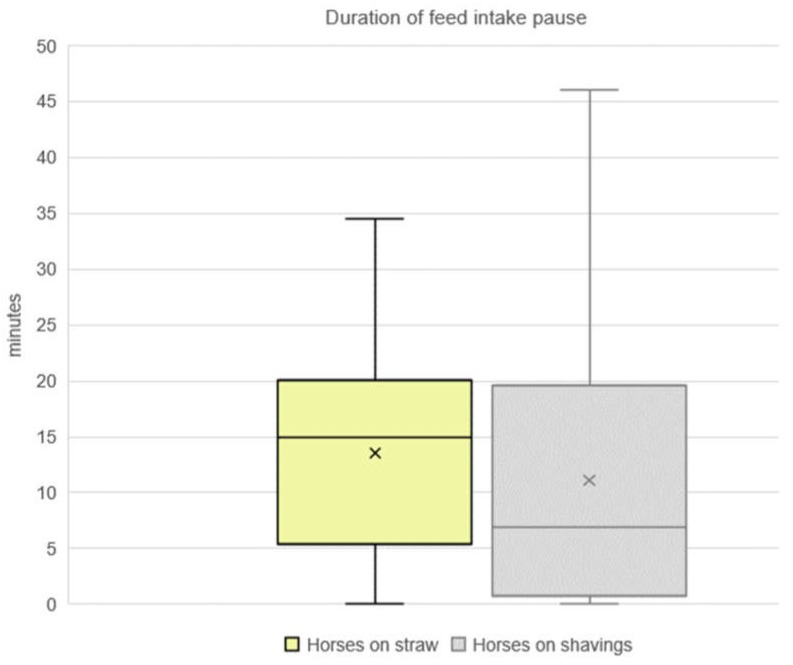
Duration of feed intake pause during the evening meal of horses on straw compared to horses on shavings (feed of hay or a mix of hay and straw to *n* = 55 horses; median: 15 min of *n* = 25 horses on straw; median: 7 min of *n* = 30 horses on shavings, n.s. with *p* = 0.36)

A similar situation was observed with regards to the duration of the feed intake pause. Horses on straw paused almost twice as long as horses on shavings (*n* = 25 horses, median: 15.0 min, mean: 14 ± 9 min versus *n* = 30 horses, median: 7 min, mean: 11 ± 12 min, n.s. with *p* = 0.36, Figure 4). The farm, specified as a random effect, influenced the duration of the feed intake pause of the CO-horses on the two bedding variants (*p* = 0.04).

All CO-horses paused their feed intake rather for a short amount of time (a few minutes) than for a longer duration (short pauses: 48% of all categories of pauses for horses on straw and 54% of all categories of pauses for horses on shavings, Table 3). Horses on shavings took less feed intake pauses of a medium (11-30 min) duration in comparison to horses on straw (29% medium pauses of horses on shavings versus 37% medium pauses of horses on straw). Only very few of the horses took long (>30 min) pauses (15%–17% of the horses on straw and horses on shavings, n = 46 horses). Horses on straw took both, short (1–10 min) pauses (after 108 ± 53 min) and medium pauses (after 121 ± 71 min) at an earlier latency than the horses on shavings, irrespective of whether their pause was short, long, or medium. The horses on shavings paused feed intake at the earliest 2 h after the start of the meal (128 ± 48 min). All of the horses on both types of bedding paused their feed intake for more than 30 min (long pauses) only after a latency of 3:24 h.

## 4. Discussion

Horses eat about 10 (7–13) meals in regular intervals within 24 h [4,7,17,55]. They hardly ever pause their feed intake for several hours [6,7,8,21,22,55]. Horses usually spend no hours without feeding [8,22]. They rarely stop foraging for longer than 3 hours (2 ± 1:18 h) [see for review 31] with 4 hours being the rare maximum [6,7,8,21,22,55]. The 24-h time budget of horses kept in individual housing systems on non-edible bedding differs greatly from that of free-ranging horses [1,5]. Kiley-Worthington [5] observed that considerable deviations in the time budget of the horses occur when housed in conditions with restrictive hay feeding and non-edible bedding. Instead of the natural feed intake duration of 12 - 16 h, she observed that an approximate feed intake of 4 hours during the 24-h day is possible at times. Especially if the feeding is restrictive and horses are housed on non-edible bedding, there is a higher risk that the maximum feed intake pause of 4 hours is exceeded [6,7,22,55]. The importance of continuous roughage intake for the physical and mental health of horses is often underestimated in practice [7,8]. The digestive system of the horses is in construction and function adapted to long feeding periods and a continuous feed intake, distributed over the 24-h day. Any major deviation from this disrupts the digestive process [36] and leads to behavioural disorders and even stereotypies (e.g., wood-chewing, ingesting wood-shavings and performing coprophagy, crib-biting, windsucking, weaving) [7,44,56,58]. A current study confirms alterations in the behaviour of horses housed in individual housing systems with restrictive feeding and non-edible-bedding. In particular, horses kept on straw express less aggressive behaviour compared to those living on non-edible bedding [58]. In our observations during the present study we noticed several times that horses on shavings showed abnormal behaviour and were aggressive towards horses on straw. This is in line with recent studies and might be indicative of the emotional state of frustration of the horses on non-edible bedding (frustration-related behaviour). Since only feed intake behaviour was the focus of the present study, no conclusions can be drawn from the observations mentioned.

We included 104 horses on 10 farms in our study of which the majority had a nocturnal feed intake interruption of 8:50 ± 1:25 h when kept on shavings. This clearly exceeds the required maximum of 4 hours according to the Guidelines to Good Animal Welfare Practice for the Keeping of Horses [55]. A horse would not take a voluntary feed intake pause of approx. 9 h because of its almost permanent motivation to forage. Therefore, feed-intake pauses of such length are not in line with the natural feeding behaviour of horses [7,22]. According to Wackenhut [63] and Mellor et al. [64] the proportion of horses kept individually on shavings in Germany and England is approximately 35%, which underlines how important it is to focus on the potential risks for animal welfare. Three basic requirements have to be fulfilled to ensure animal welfare: basic health and functioning, natural living (behaviour) and affective states [54]. If the horses´ feed intake pauses are too long, the horses are not able to act out their natural behaviour, which potentially compromises their welfare [54]. Furthermore, health problems in the digestive tract, i.e., grade II gastric ulcers, occur considerably more often if feed intake pauses last for more than 6 hours [37].

Only on two farms all of the horses on non-edible bedding still had roughage available at midnight. If the horses on these two farms would carefully divide the remaining quantity of roughage (an amount for approximately 1:30 h of feed intake time), they would have been able to take a feed intake pause of a maximum of 4 hours [6,55]. However, this could not be reliably assessed, as the observations had to stop at midnight for operational constraints. If the nocturnal feed intake interruption of all horses on non-edible bedding should be determined, the observation would have had to be continued during the nighttime, because 25.68% of horses did not finished their evening meal by midnight (*n* = 19/74).

All of the horses were allowed to graze daily. The grazing period ended at least 2 h prior to the evening feeding. Hence, it seems that the availability of straw predominantly determines whether the feed intake behaviour of horses on non-edible bedding differs from that on edible bedding (less time spent on the evening meal and fewer feed intake pauses). We observed that one horse bedded on straw preferred to forage straw instead of hay although hay was still available. If such a preference for straw would have been observed in more horses, we would have included it as a possible influence factor on the longer overall feeding time for the evening meals of the horses on straw in comparison to the horses on shavings. Since this happened only in one horse and only for a limited period, we can exclude this as a bias in the results.

Since we tried to bias the feeding routine as little as possible and did therefore not measure the specific hay amount for the calculation of the feed intake rate, we can only draw an indirect conclusion on the accelerated feed intake rate from the horses’ modified feed intake behaviour on non-edible bedding. It can be assumed that horses on shavings ate their meal at a much faster rate, since it took them less time to finish the evening meal than the horses housed on straw. In addition, the former took significantly fewer feed intake pauses and only at a later latency. This indicates to an accumulated need deficit that could possibly lead to a “rebound effect” in horses on non-edible bedding. Accelerated feed intake due to lack of roughage or shortened grazing periods is in line with previous studies [8,65,66]. It has already been shown that horses suffer from a “rebound effect” as mentioned above when they are unable to act out their natural behaviour such as locomotion or resting [67,68,69,70]. Altered feed intake behaviour of horses on shavings as a consequence of restrictive roughage supply indicates an impaired welfare.

The treatment groups lacked uniformity, which could be considered as a restraining factor for the study. Our goal was to include a representative number of horses on ten different farms in order to obtain valid results concerning feeding practices for individually housed horses. We did not preselect the horses in advance, which might have biased the results. We recorded the end of the evening meal and the beginning of the morning feeding for 40 additional horses on non-edible bedding and were thus able to increase the sample size for the present study. Moreover, it was inevitable that horses were taken away for equestrian use during the observation periods. For this reason, we separated the horses in two groups: One continuously observed group and one discontinuously observed group. The confounding factor “equestrian use” applied to the latter. We decided that the temporary absence of the horse during the evening meal might have an influence on the natural feed intake behaviour, especially on the feed intake pauses. We excluded these horses (n = 9) in the analysis of the feed intake behaviour. For the stated reasons, not all hypotheses could be assessed on the basis of the exact same sample size of horses. Current welfare assessment protocols for horses measure the welfare criterion of “good feeding” or the welfare parameter of “freedom from hunger” only against the indicator “body condition score” [71,72,73]. However, this indicator can only be used to evaluate if the horses´ nutritional status is adequate. To date, current welfare assessment protocols for horses [54,55,71,73] do not take the indicators into account that relate to the behavioural and physiological aspect of appropriate feeding. In order to respect the psychological and physical well-being of the horses, one has to be aware of the fact that the feed intake behaviour is a basic high priority need of the horse. Excessive feed intake pauses as found in the present study represent a risk of pain, suffering and harm [5,18,21,33,37,42,51]. For this reason, a German court confirmed in its judgement (2019, file number RN 4 K 17.1298, Regensburg Administrative Court) that it has to be ensured that the maximum duration of feed intake pauses is 4 hours and that the duration of feed intake must at least be 12 h.

## 5. Conclusions

We conclude from our study that common feeding practices (two or three roughage meals during daytime) impair the horses´ welfare if they are kept on non-edible bedding. The reason for this is that in such housing systems the majority of the horses pause their feed intake for too long during the night. The average nocturnal feed intake interruption of horses observed in individual housing system on non-edible bedding was 9 h. Thus, the maximum tolerable feed intake pause of 4 hours is exceeded. Consequently, the well-being of the horses might be at risk. The changed feed intake behaviour of the horses allows us to draw a similar conclusion. The horses on non-edible bedding finished their hay meal faster and took fewer pauses during the meal than horses on edible bedding (“rebound effect”). If the basic high priority need of the horse to continuously engage in feed intake is neglected and the horses’ access to roughage (straw) is limited, the horses will accelerate their feed intake of the roughage and eat almost non-stop. Altered feed intake behaviour indicates an impaired welfare.

However, further studies should be carried out in order to assess additional behavioural and physiological effects of restrictive feeding practices when there is no additional straw available. Video surveillance or longer observation periods could have helped to find out if all horses on non-edible bedding were affected by prolonged feed intake interruptions. Nevertheless, restrictive feeding of roughage for horses kept on non-edible bedding such as shavings or straw pellets can be regarded as inadequate. Novel feeding techniques (e.g., supply of roughage via forage feeding systems such as automated roughage feeders, feeding racks, time-controlled automatic feeders) need to be implemented and could satisfy the behavioural needs. Future welfare assessment should take the duration of the feed intake pauses into account. The “Weihenstephan’s evaluation system for the assessment of animal welfare and the environmental impact of horse husbandry”, which is currently under development, shall be used as a scientific consultation tool in Germany in the future. The tool includes the holistic feeding aspects of horses.

## Figures and Tables

**Table 1:** Classification of horses per bedding type and observation method.

Category	CO-Horses ^2^	DCO-Horses ^3^	AR-Horses ^4^	Total
Horses on straw	25	5	-	30
Horses on shavings	30 (25) ^1^	4 (4)^1^	40 (26) ^1^	74 (55) ^1^
Total	55	9 (9)^1^	40 (26) ^1^	104 (90) ^1^

^1^ Number of horses that finished their evening meal within the observation period ^2^ Continuously Observed horses; ^3^ DisContinuously Observed horses; ^4^ Additionally Recorded horses.

**Table 2:** Number of Continuously Observed (CO-) horses per farm and condition (bedding material: straw and shaving).

Farm	Horses on Straw Per Farm	Horses on Shavings Per Farm	CO-Horses in Total
1	4	6	10
2	2	5	7
3	3	1	4
4	3	2	5
5	3	3	6
6	3	6	9
7	7	2	9
8	0	1	1
9	0	3	3
10	0	1	1
Total	25	30	55
Min	0	1	1
Max	7	6	10
Mean	2.5	3	5.5
Median	3	2.5	5.5

**Table 3:** Latency from the start of the evening feeding until the first pause divided into three categories according to the duration of the feed intake pauses (mean ± SEM, in minutes) and frequency of pauses per each category for horses on straw and shavings (in %).

Category	Short Pauses (1–10 Min)		Medium Pauses (11–30 Min)		Long Pauses (> 30 Min)	
**Sample Size (Number of Horses)**	**55**		**55**		46 ^1^	
Horses on straw	48% ^1^	108 ± 53	37% ^1^	121 ± 71	15% ^1^	204 ± 67
Horses on shavings	54% ^1^	128 ± 48	29% ^1^	168 ± 79	17% ^1^	204 ± 86

^1^*n* = 9 horses that did not take long pauses were removed from the calculation of frequency of pauses per category (in %).

## Supplementary Materials

The following are available online at https://www.mdpi.com/2076-2615/10/3/411/s1, Table S1: Table S1: Total time for finishing the evening meal dependent on farm and bedding; Table S2: Total time for finishing the evening meal dependent on beddingfor feed intake behaviour analysis. Table S3: "Horses on shavings“, which ate up their evening meal within the observation period (Calculation of the duration of the nocturnal feed intake interruption; Table S4: Frequency of pauses, duration of pauses and latency until first feed intake pause during evening meal; Table S5: Number of feed intake pauses dependent on bedding.
