# Supplementary material for: Common Feeding Practices Pose A Risk to the Welfare of Horses When Kept on Non-Edible Bedding
Source: Animals (Basel). 2020 Mar 2;10(3):411. doi: 10.3390/ani10030411 (PMC7142811; doi:10.3390/ani10030411)
Supplement: Supplementary file 1 [file animals-10-00411-s001.pdf]

Table S1: Total time for finishing the evening meal dependent on farm and bedding

Farm	Bedding	Duration in minutes
1	shaving	320
1	shaving	334
1	shaving	334
1	shaving	210
1	shaving	290
1	shaving	320
2	shaving	237
2	shaving	312
2	shaving	269
2	shaving	238
2	shaving	206
2	shaving	310
2	shaving	306
2	shaving	307
3	straw	360
3	straw	310
3	straw	344
3	straw	343
3	shaving	323
3	straw	313
3	shaving	358
4	shaving	273
4	shaving	132
4	straw	277
4	straw	237
5	shaving	400
5	shaving	182
5	shaving	363
5	straw	209
5	straw	336

5	straw	420
7	shaving	333
7	shaving	333
7	shaving	177
7	shaving	239
7	shaving	333
7	shaving	195
7	shaving	263
8	shaving	371
8	shaving	100
8	straw	339
8	shaving	135
8	straw	363
8	straw	325
9	straw	351
10	shaving	247
10	shaving	300
10	shaving	271
10	straw	270
10	straw	235
10	straw	238

Table S2: Total time for finishing the evening meal dependent on bedding

Time for finishing the evening meal for horses on straw (in min)	Time for finishing the evening meal for horses on shavings (in min)
360	320
310	334
344	334
343	210
313	290
277	320

	237	237
	209	312
	336	269
	420	238
	339	206
	363	310
	325	306
	351	307
	270	323
	235	358
	238	273
		132
		400
		182
		363
		333
		333
		177
		239
		333
		195
		263
		371
		100
		135
		247
		300
		271
**Median**	**325**	**307**

Table S3: "Horses on shavings", which ate up their evening meal within the observation period (Calculation of the duration of the nocturnal feed intake interruption)

Count of CO and DCO-horses	Farm	horse per farm	Beginning of evening feed	End of evening feed	Beginning of morning feed	Nocturnal feed intake pause
1	1	Shavings Boogie	16.45	22.05	05.45	460
1	1	Shavings Skippa	16.45	22.19	05.45	446
1	1	Shavings Snappy	16.46	20.16	05.45	632
1	1	Shavings Charly	17.00	21.50	05.45	475
1	1	Shavings Henry	17.00	22.20	05.45	445
1	1	Shavings Chestnut	17.00	23.00	05.45	405
1	2	Shavings Colinda	18,03	22.00	7.15	555
1	2	Shavings Alisha	18.04	22.33	7.15	522
1	2	Shavings Mistou	18.10	22.08	7.15	547
1	2	Shavings Stanley	18.04	21.30	7.15	585
1	2	Shavings Dilano	18.05	23.15	7.15	480
1	3	Shavings Whoopi	16.47	22.10	6.55	525
1	3	Shavings Lukas	16.47	22.45	6.55	490
	3	Shavings Piccolina	16.40	22.40	6.55	495
1	4	Shavings Dun horse	17.22	21.53	05.10	437
1	4	Shavings Gray horse	17.22	20.30	05.10	520
	4	Shavings Fleabitten grey horse I	17.29	21.10	05.10	480
	4	Shavings Bayard	17.28	20.46	05.10	456
	4	Shavings Fleabitten grey horse II	17.25	21. 58	05.10	432
1	5	Shavings Cindy	15.30	22.10	7.00	539
1	5	Shavings horse left	17.15	20.17	7.00	643
1	5	Shavings horse rech	16.27	22.30	7.00	510
	5	Shavings Chestnut	15.30	17.37	7.00	803
	5	Shavings Barry	15.40	18.40	7.00	740

	5	Shavings Fleabitten grey	15.40	22.00	7.00	540
1	6	Shavings Double Beat	17.17	22.50	7.00	530
1	6	Shavings Promi	17.17	20.14	7.00	646
1	6	Shavings Raika	17.17	21.16	7.00	584
1	6	Shavings Carlos	17.17	22.50	7.00	530
1	6	Shavings Vivien	17.17	20.32	7.00	688
1	6	Shavings Manni	17.17	21.40	7.00	560
	6	Shavings Henry	17.15	22.00	7.00	540
	6	Shavings Nala	17.13	22.30	7.00	510
	6	Shavings Raminia	17.15	21.30	7.00	570
	6	Shavings Whisky	17.13	22.00	7.00	540
	6	Shavings Dihlara	17.13	22.00	7.00	540
1	7	Shavings Clyde	17.04	23.15	6.30	435
1	7	Shavings Sunny	17.05	18.45	6.30	705
1	7	Shavings Candy Girl	17.04	19.19	6.30	671
	7	Shavings Cassandra	17.13	20.26	6.30	614
	7	Shavings Pellegrino	19.00	23.30	6.30	420
	7	Shavings Rosco	19.00	21.30	6.30	540
	7	Shavings Zelela	17.14	22.05	6.30	445
	7	Shavings Heron	17.24	22.05	6.30	445
	7	Shavings Ramira	17.24	21.10	6.30	560
	7	Shavings Lupino	17.18	21.05	6.30	565
	7	Shavings Askadron	17.14	20.55	6.30	575
	7	Shavings Kalimero	17.23	22.05	6.30	445
1	8	Shavings Luna	17.03	21.10	5.30	500
1	8	Shavings Souris	16.59	21.30	5.30	480
	8	Shavings Angelo	16.52	22.00	5.30	450
	8	Shavings Finale	16.51	21.45	5.30	465
	8	Shavings Cara Donna	17.00	22.00	5.30	450
	8	Shavings Jaguar	16.42	22.10	5.30	440

	8	Shavings Petja	16.42	20.31	5.30	539
**29**		**CO and DCO-horses**				
**26**		**AR-horses**				
		Mean				529.89
		Standard deviation				85.38
		Median				525
		Min				405
		Max				803
		N				55

Table S4: Frequency of pauses, duration of pauses and latency until first feed intake pause during evening meal

Farm	Bedding	Horse	Latency until the first feed intake pause (in min)	Number of feed intake pauses (n)	Duration of feed intake pause (in min)
1	shaving	shaving 1	109	1	1
1	shaving	shaving 2	334	0	0
1	shaving	shaving 3	169	5	6.4
1	shaving	shaving 4	157	1	1
1	shaving	shaving 5	290	0	0
1	shaving	shaving 6	70	3	19.3
1	straw	straw 1	62	1	15
1	straw	straw 2	33	3	16
1	straw	straw 3	47	4	25
1	straw	straw 4	134	3	5
2	shaving	shaving 7	164	3	12.3
2	shaving	shaving 8	71	7	6.4
2	shaving	shaving 9	167	4	6.5
2	shaving	shaving 10	71	3	20.3

2	shaving	shaving 11	251	1	46
2	straw	straw 5	149	3	3.3
2	straw	straw 6	152	1	1
3	shaving	shaving 12	35	3	31.7
3	straw	straw 7	26	3	3.7
3	straw	straw 8	336	0	0
3	straw	straw 9	420	0	0
4	shaving	shaving 13	231	3	5
4	shaving	shaving 14	132	0	0
4	straw	straw 10	87	8	15.4
4	straw	straw 11	95	4	19.5
4	straw	straw 12	157	7	5.7
5	shaving	shaving 15	70	5	7.2
5	shaving	shaving 16	120	1	1
5	shaving	shaving 17	363	0	0
5	straw	straw 13	45	4	17
5	straw	straw 14	104	12	10.8
5	straw	straw 15	70	16	7.6
6	shaving	shaving 18	123	5	18.2
6	shaving	shaving 19	83	6	20.5
6	shaving	shaving 20	158	1	9
6	shaving	shaving 21	123	4	6
6	shaving	shaving 22	195	0	0
6	shaving	shaving 23	231	1	12
6	straw	straw 16	135	2	34.5
6	straw	straw 17	132	4	7
6	straw	straw 18	223	1	10
7	shaving	shaving 24	100	0	0
7	shaving	shaving 25	135	0	0
7	straw	straw 19	126	5	22.6
7	straw	straw 20	89	5	21.6

7	straw	straw 21	46	9	20.1
7	straw	straw 22	162	3	20
7	straw	straw 23	127	4	26.3
7	straw	straw 24	56	8	14.4
7	straw	straw 25	104	6	17.5
8	shaving	shaving 26	67	8	12.9
9	shaving	shaving 27	89	3	28.3
9	shaving	shaving 28	169	2	9
9	shaving	shaving 29	111	4	24.3
10	shaving	shaving 30	203	4	28.5

Table S5: Number of feed intake pauses dependent on bedding

Horse	Bedding	Feed intake pause (n)	
1	shaving	0	No feed intake pause = 0 (7/30 horses on shavings; 2/25 horses on straw)
2	shaving	0	One or more feed intake pauses = 1
3	shaving	0	
4	shaving	0	
5	shaving	0	
6	shaving	0	
7	shaving	0	
8	shaving	1	
9	shaving	1	
10	shaving	1	
11	shaving	1	
12	shaving	1	
13	shaving	1	
14	shaving	1	

15	shaving	1
16	shaving	1
17	shaving	1
18	shaving	1
19	shaving	1
20	shaving	1
21	shaving	1
22	shaving	1
23	shaving	1
24	shaving	1
25	shaving	1
26	shaving	1
27	shaving	1
28	shaving	1
29	shaving	1
30	shaving	1
31	straw	0
32	straw	0
33	straw	1
34	straw	1
35	straw	1
36	straw	1
37	straw	1
38	straw	1
39	straw	1
40	straw	1
41	straw	1
42	straw	1
43	straw	1
44	straw	1
45	straw	1
46	straw	1

47	straw	1
48	straw	1
49	straw	1
50	straw	1
51	straw	1
52	straw	1
53	straw	1
54	straw	1
55	straw	1
